# Oxygen Selective Membranes for Li-Air (O_2_) Batteries 

**DOI:** 10.3390/membranes2020216

**Published:** 2012-05-11

**Authors:** Owen Crowther, Mark Salomon

**Affiliations:** MaxPower, Inc., 141 Christopher Lane, Harleysville, PA 19438, USA

**Keywords:** lithium-air battery, oxygen selective membrane, oxygen permeability, ambient discharge

## Abstract

Lithium-air (Li-air) batteries have a much higher theoretical energy density than conventional lithium batteries and other metal air batteries, so they are being developed for applications that require long life. Water vapor from air must be prevented from corroding the lithium (Li) metal negative electrode during discharge under ambient conditions, *i.e*., in humid air. One method of protecting the Li metal from corrosion is to use an oxygen selective membrane (OSM) that allows oxygen into the cell while stopping or slowing the ingress of water vapor. The desired properties and some potential materials for OSMs for Li-air batteries are discussed and the literature is reviewed.

## 1. Introduction

**Figure 1:**
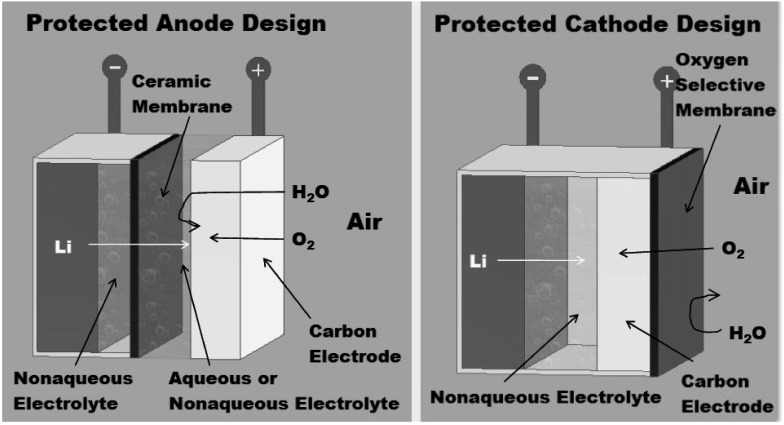
Designs for Li-air batteries. In the protected anode approach shown at the left, the lithium anode is protected by a glass-ceramic disc (membrane) which is hermetically sealed to the anode. This solid-state glass-ceramic is a Li^+^ conductor impervious to water. In the protected cathode design shown at the right, the glass-ceramic solid-state membrane is removed and instead a flexible OSM is attached to the surface of the air electrode facing the atmosphere.

While metal-air batteries based on zinc, magnesium and aluminum anodes are well developed technologies [1], the development of lithium-air (Li-air) batteries had remained elusive until the breakthrough study by Abraham and Jiang [2]. The attractiveness of Li-air batteries compared to other common battery systems is due to the promise of greatly improved energies and capacities for Li-air batteries. Theoretical energies and capacities for selected electrochemical systems were calculated [3,4,5] from critically evaluated Gibbs energies of formation data from [6], and the results are given in Table 1. The increasing number of publications on Li-air batteries is indicative of the importance of these batteries for future applications to electric vehicles (EVs) and hybrid electric vehicles (HEVs), portable electronics, unattended remote sensors and other applications that have recently been reviewed by Christensen *et al*. [7] and by Crowther and Salomon [8]. For the present review, we focus on new approaches under development at MaxPower and others relating to oxygen selective membranes (OSMs) for the air electrode of a primary nonaqueous, Li-air battery. Figure 1 shows two Li-air designs presently under development at MaxPower.

**Table 1:** Theoretical specific energy and capacity comparisons for selected battery systems.

Metal-air system	OCV (V)	Specific energy (Wh/kg)	Specific capacity (mAh/g)
2Li + ½O_2_→ Li_2_O (aprotic organic sln)	2.913	11,248 *	3,862
Li + ½O_2_ ⇌ ½Li_2_O_2_ (aprotic organic sln)	2.959	11,425 *	3,862
2Li + ½O_2_ + H_2_SO_4_ ⇌ Li_2_SO_4_ + H_2_O	4.274	2,046 *	479
2Li + ½O_2_ + H_2_O ⇌ 2LiOH	3.446	5,789 *	1,681
2Li + H_2_O (seawater) → 2LiOH + ½H_2_	2.512	9,701 **	3,862
Al + 0.75O_2_ + 1.5H_2_O ⇌ Al(OH)_3_	2.701	4,021 *	1,489
Mg + ½O_2_ + H_2_O ⇌ Mg(OH)_2_	2.756	3,491 *	1,267
Zn + ½O_2_ ⇌ ZnO	1.650	1,353 *	820
x6C + LiCoO ⇌ xLiC_6_ + Li_1-x_CoO_2_ (organic)	~4.2	420 ***	139 ***

* The molecular mass of O_2_ is not included in these calculations because O_2_ is freely available from the atmosphere and therefore does not have to be stored in the battery or cell; ** The molecular mass of H_2_O is not included since it is freely available from seawater (pH 8.2) and does not have to be stored in the battery. *** Based on x = 0.5 in Li_1−x_CoO_2_.

The design for the protected anode model is dependent upon a lithium ion (Li^+^) conducting membrane which is impervious to water, and for both aqueous and nonaqueous electrolyte solutions, present research for this membrane is focusing on Li^+^ conducting glass ceramics such as those developed by O’Hara Corporation [9,10,11] and its use in practical systems as originally proposed by Visco *et al*. [12]. Li-air cells based on the protected anode are well suited for application to both primary and rechargeable batteries, but technical problems relating to membrane chemical and mechanical stability, high cost and high resistivity are presently key areas of R&D as reviewed recently by Christensen *et al*. [7] and Crowther and Salomon [8]. In addition, secondary Li-air batteries face the problem of Li dendrite formation during charging that will limit cell cycle life and safety [13]. For the present review, we focus on nonaqueous primary Li-air cells based on the protected cathode design that use OSMs as shown in Figure 1.

## 2. Properties of Oxygen Selective Membranes

The protected cathode design for a Li-air power source is best suited for primary cells and batteries using nonaqueous electrolyte solutions. An OSM for Li-air cells and batteries should possess the following desired properties that are discussed in greater detail below.

1. High O_2_ permeability;
2. No water vapor permeability;
3. No electrolyte solvent permeability;
4. No carbon dioxide (CO_2_) permeability for secondary applications.

### 2.1. High O_2_ Permeability

Basic materials for use as OSM must be capable of dissolving large amounts of O_2_ (oxygen) and possess a high rate at which O_2_ can diffuse through these membranes. The permeability, 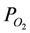, or transmission rate of O_2_ (OTR) through a barrier is dependent upon the solubility of O_2_ in the membrane and the rate of diffusion of O_2_ through the membrane, and is defined by

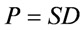
(1)
where *D* is the diffusion coefficient [cm^2 ^s^−1^] and *S* is the solubility coefficient (not solubility). The solubility coefficient can be determined experimentally or from known values of Henry’s Law constants, *K_H,cp_*, defined as

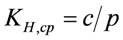
(2)
where *c* is the concentration [mole L^−1^] and *p* is the partial pressure of O_2_ [mm Hg or Pa]. For most nonaqueous electrolyte solutions used in Li-air cells, *K_H,cp_* data for O_2_ are not available, but for a number pure nonaqueous solvents, *K_H,cp_*, values and solubilities have been compiled by Battino [14,15] and Dias *et al.* [16]. The units for permeability, *P*, normalized for membrane thickness is, from Equation (1).

*P =* cm^3^ (gas) cm cm^−2^s^−1^ mm Hg^−1^ (3)

where cm^3^ (gas) is actually a molar quantity evaluated at standard temperature and pressure that can determined from *K_H,cp_* data. The units for *P* are often given in Barrer which is defined by

1 Barrer = 10^−11^ cm^3^ (gas) cm cm^−2^ mm Hg^−1^ (4)

The oxygen flux, 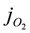, can be directly related to maximum (limiting) current density, *i_max_*, assuming the oxygen reduction reaction (ORR) is mass transfer limited by O_2_ transport through the OSM. The maximum (limiting) current density due to O_2_ diffusion for an air cathode protected by an OSM is highly dependent on the magnitude of *P*, and below we show that membranes where *P* < 100 Barrer are not practical for a commercial Li-air cell. In view of the limited amount of data required to calculate permeability values from Equations (1–3), values of *P* can easily be determined experimentally using a barrier testing instrument such as the one available from MOCON, Inc.

Abraham and Jiang [2] originally hypothesized that the oxygen reduction reaction (ORR) in nonaqueous electrolyte solutions proceeds by the following mechanisms during discharge of a Li-air battery.


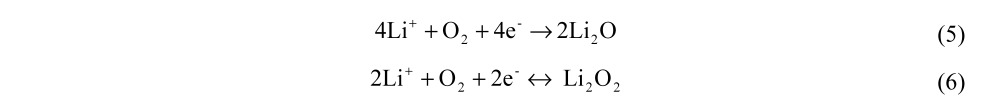


Several recent publications, reviewed by Christensen *et al.* [6], demonstrated that the solvent actually reacts during ORR for electrolytes that use alkyl carbonates as solvents to form products with limited reversibility, namely lithium carbonate and lithium alkyl carbonates, and not the desired lithium peroxide. However, ORR in ether-based electrolyte solutions will proceed predominantly according to Equation (6). 

**Figure 2:**
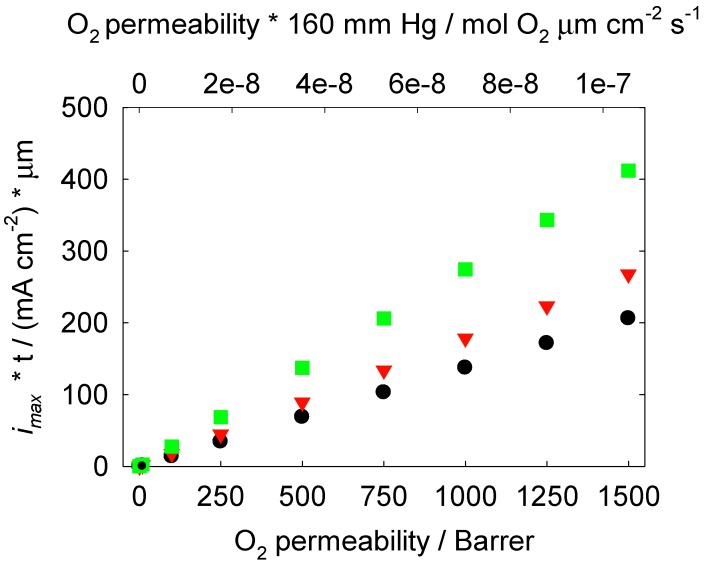
Effect of O_2_ permeability on maximum current density, *i_max_*, for a 1 µm thick OSM. Black circle points correspond to n = 2, red triangle points correspond to n = 2.6, green square points correspond to n = 4 equivalent e^−^ per mole of O_2_.

The maximum current density due to diffusion of O_2_ through the membrane is based on the oxygen flux, 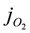, which can be related to permeability by the following equation. 


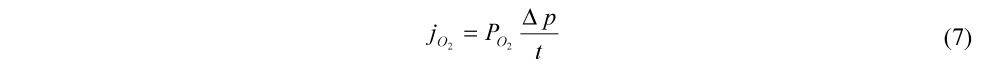

where 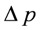 is the partial pressure gradient of O_2_ across the membrane and *t* is the membrane thickness. The partial pressure of O_2_ in air is ~160 mm Hg and will be 0 in the air electrode assuming O_2_ is the limiting reactant so 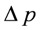
= 160 mm Hg. The flux of O_2_ through the membrane can also be improved by decreasing the thickness of the membrane. However, membrane fabrication techniques typically limit the thickness to 10–25 µm before pinhole defects are present. Also, decreasing the thickness of the membrane will also increase the permeation of water vapor and electrolyte solvent. The maximum current density is given by

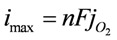
(8)
where *n* is the equivalents of electrons reacted per mole of oxygen and *F* is Faraday’s constant 
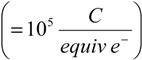
. From Equations (5) and (6), *n* is equal to 4 for Li_2_O formation and 2 for Li_2_O_2_ formation. McCloskey *et al.* [17] experimentally determined n = 2.6 for the ORR in alkyl carbonates due to the reaction of the electrolyte solvent with Li^+^ and O_2_ to form lithium carbonate and lithium alkyl carbonates. Figure 2 shows the effect of O_2_ permeability in Barrer on maximum current density for a 1 µm thick membrane. The data were derived using the Equations (3, 4, 7, 8). This assumes the air window and air electrode have equal geometric areas. The maximum current density for a Li-air battery limited by O_2_ transport through an OSM of any thickness can be determined by dividing the i_max_ for a given permeability obtained from Figure 2 by the actual thickness in microns of the OSM. For example, a membrane that is 1 µm thick with a permeability of 500 Barrer would have a limiting current density of approximately 68 mA cm^−2^ based on Li_2_O_2_ formation. The limiting current density is 1.4 mA cm^−2^ for the same membrane if it is 50 µm thick.

### 2.2. No Water Vapor Permeability

**Figure 3:**
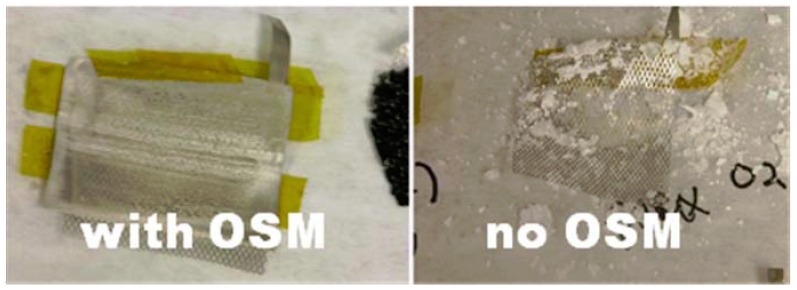
Pictures of Li metal negative electrode after cell discharge in humid air for cells using an OSM (left) and with no membrane (right).

The three important gases in dry air are N_2_ (78 o/v), O_2_ (21 o/v) and CO_2_ (0.4 o/v), water being variable at ~1–4 o/v depending upon relative humidity. Materials considered for membranes will vary in ability to dissolve and transmit these gases. A solid, gel or immobilized liquid is required to maximize O_2_ transmission and retard water vapor. Since the molecular diameter of water is smaller than that of oxygen, porous membranes that separate gases based on Knudsen diffusion will not work for this application. In fact it is extremely difficult to prevent water transmission through membranes optimized for high O_2_ permeabilities. Figure 3 shows the Li metal negative electrode from cells discharged in wet air (23 °C, 20% RH) using a Teflon coated fiberglass cloth (TCFC) OSM (left) and without OSM [18]. The Li is relatively pristine for the cell that used the OSM and completely consumed to form lithium hydroxide in the cell with no OSM. 

Practically, it is not necessary for an OSM to be a perfect water vapor barrier for primary applications. However, small amounts of water will react with Li to form a resistive surface film. Even water concentrations of 200 ppm will increase the Li resistivity, most likely due to thickening of the film on the Li. This exact increase in resistivity is highly dependent on electrolyte salt, electrolyte solvent, and the amount of water in the electrolyte [19]. MaxPower has found that selectivities of 2.5 to 5 mol O_2_ per mol H_2_O will allow for normal discharge of a primary Li-air cell for at least one week.

### 2.3. No Electrolyte Solvent Permeability

**Figure 4:**
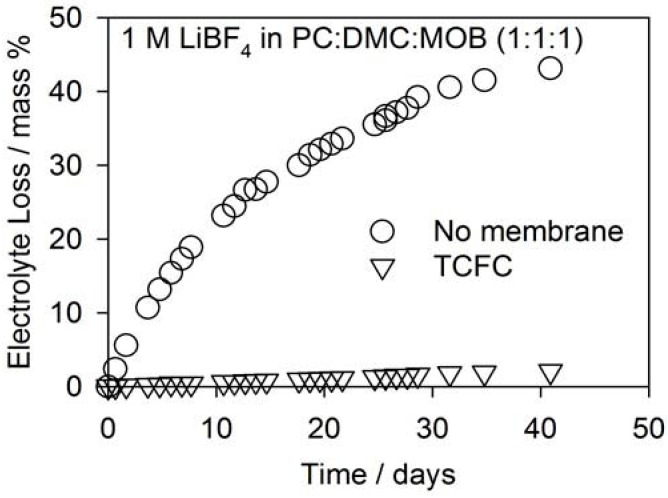
Evaporation rates of a typical electrolyte from a pouch cell with and without an OSM [18].

Read *et al.* [20] demonstrated increasing the O_2_ solubilities of a Li-air electrolyte results in increased capacity. However, electrolyte solvents with high O_2_ solubilities tend to have low viscosities and boiling points and will evaporate quickly [20,21]. Electrolyte evaporation will lead to premature cell failure since Li^+^ in the electrolyte will not be able to reach all of the active carbon reaction sites. Figure 4 shows the evaporation rate for a typical lab cell with and without an OSM [16]. The electrolyte solution used in this study was 1 mol dm^−3^ LiBF_4_ in a 1:1:1 (o/v) solvent mixture of propylene carbonate (PC), dimethyl carbonate (DMC) and methoxybenzene (MOB). The use of a TCFC OSM reduces the evaporation from approximately 40% to 2% over the course of a month, a very acceptable number for continuous operation for primary applications. MaxPower has observed materials with high water vapor permeabilities also have high electrolyte solvent permeabilities. One measure of electrolyte compatibility is to soak the OSM in a given electrolyte for a period of time. Ideally, there would be no mass gain and no swelling of the OSM after removal from the solvent. The actual solvent permeation rate can also be measured according to ASTM F739.

### 2.4. No CO_2_ Permeability for Secondary Applications

Carbon dioxide transmission through most OSM materials is often comparable to oxygen where, for example, ratios of *P*(O_2_)/*P*(CO_2_) are often less than unity. Carbon dioxide will react with the Li_2_O_2_ (or even Li_2_O) discharge product to form lithium carbonate (Li_2_CO_3_). Li_2_O_2_ can reversibly evolve O_2_ during cell charging while Li_2_CO_3_ cannot [22]. McCloskey *et al*. [23] recently highlighted additional problems with Li_2_CO_3_ formation. Therefore, the OSM, if being used for a rechargeable Li-air battery, must also be an effective CO_2_ barrier for acceptable cycle life. This is a challenge since materials with high O_2 _permeabilities tend to also have high CO_2_ permeabilities [14,15,16]. However, the actual discharge product does not matter in primary Li-air cells so there is no requirement that the OSM is a CO_2_ barrier for primary applications [18].

Nitrogen being inert will also have little or no effect on the performance of Li-air cells. It is interesting to note the for materials which exhibit high O_2_ solubility, *P*(O_2_)/*P*(N_2_) ratios are generally >1 thus slightly enriching the relative O_2_ content over N_2_.

## 3. Basic Materials for Oxygen Selective Membranes

Oxygen selective membranes can be fabricated or synthesized, or commercially obtained as polymeric films. Two specific (preferred) groups of materials of interest for fabrication of O_2_-selective membranes in various configurations (gels, polymers, liquids immobilized in a porous inert substrate) include the following.

1. Fluorinated hydrocarbons, polyethers (e.g., Krytox 1506), polyperfloroalkyl oxides, (polyperfloroalkyl amines) have been used membranes based on liquids immobilized in inert substrates [24,25,26], gels [26,27], and chemically and UV cured polypefloroalkyl oxides [25]. Typical values for *P*(O_2_) for these membranes approach 1000 Barrer with *P*(O_2_)/*P*(H_2_O) ~3–4 [25].

2. Polysiloxanes, silicone oils, fluorinated polysiloxanes, and fluorinated polysiloxane copolymer with alkyl methacrylates. Polysiloxanes are thermally stable, exhibit high O_2_ solubilities, can be used as a liquid immobilized in an inert polymer [25] and thermally or UV cured (vulcanized) to produce a silicon rubber [4,25,26,27] Typical values for the permeability of O_2_ in dimethylsilicone rubbers are above 600 Barrer [28,29], and around 100–250 Barrer in methacryloxypropyl terminated polydimethyl contact lenses [30]. 

Other materials that have been tested as an OSM for Li-air batteries are Melinex 301H (ML) [31,32,33], high density polyethylene (HDPE) [31], silicalite zeolite or PTFE on nickel foam support [34], silicone oil immobilized in Teflon (PTFE) or nickel/ytrria stabilized zirconia (YSZ)/silicate layered membranes [35], and TCFC with [36] and without [18,36] a silicone adhesive layer. These materials will all be discussed further in below. 

## 4. Review of Oxygen Selective Membrane Literature

The two most active groups of researchers developing OSMs are those from MaxPower Inc. and the Pacific Northwest National Laboratory (PNNL). Both groups report different experimental approaches, and a concise summary of these approaches is presented here. 

Zhang *et al*. [31] tested both ML and HDPE in various sized Li-air cells discharged under ambient conditions. ML (Dupont Teijin Films) is a heat sealable polymer that acted both as the packaging and OSM. It consists of a polyethylene terephthalate (PET) layer and a terephthalate/isophthalate copolyester of ethylene glycol thermal bonding layer. Cells using 20 µm ML were able to discharge in 20% RH for 33 days at a current density of 0.05 mA cm^−2^ with a taper at the 2V cutoff to 0.01 mA cm^−2^. An optimized pouch cell demonstrated a energy density of 362 Wh kg^−1^ including package, which is the highest published energy density for a nonaqueous Li-air battery. Cells using 30 µm thick ML could not discharge for more than one day due to O_2_ starvation. The measured O_2_ permeability of the ML membrane was ~0.02 Barrer. This value corresponds well with the literature value of PET of 0.01 Barrer [37]. From this permeability value the maximum operating current density for this cell ~0.2 µA cm^−2^ depending on the discharge product using the Equations (4), (7) and (8). The cell packaging was made of ML, so the assumption in Equation (8) that the OSM and air electrode have equal areas is not true in the case. Applying an assumed correction factor that the OSM has an area of 3 times the electrode, the cell should only be able to support a current density of ~0.6 µA cm^−2^. This is two orders of magnitude lower than the actual current density of 50 µA cm^−2^. This discrepancy was attributed to the fact the ML absorbed electrolyte causing the polymer pore size to expand resulting in a higher diffusion coefficient for O_2_ than in the case of the dry measurement of permeability. Higher O_2_ diffusion coefficients would result in higher permeabilities via Equation (1). The low current density results in a lower power density of 46 mW g^−1^ that limits the applications of this battery despite the high energy density [38]. HDPE was also tested in this study but the cells did not run well despite the improved O_2_ permeability of 2 Barrer (membrane from Mid South Extrusion) and 4 Barrer (membrane from Blueridge Films, Inc.). This is most likely because the permeabilities of moisture was also high, so the cells failed because of corrosion of the Li anode [31].

Other OSM membranes developed at PNNL were supported either YSZ/silicate or PTFE on Ni metal foam substrates [34]. Initial experiments on these membranes proved unsuccessful because the porosity of the membranes allowed water vapor to enter the cell via Knudsen diffusion and corrode the Li anode. The cells performed better when a dense, thin PTFE membrane was spin coated on the substrate. Cells using this membrane were able to discharge for 21 days in air at 20% RH at a current density of 0.05 mA cm^−2^, corresponding to capacities of 1,022 mAh g^−1^ C. The control cell for this experiment discharged under the same conditions with no OSM only demonstrated a capacity of ~300 mAh g^−1^ C. The actual thickness of the PTFE layer was 1–10 µm. The literature value for O_2_ permeability in Teflon is 4–10 Barrer [39]. Therefore, a 1 µm membrane should be able to support a 0.25–2 mA cm^−2^ discharge depending on the actual permeability and discharge product. Thicker membranes would demonstrate lower maximum current densities. However, they did not attempt to test these membranes at higher rates, to our knowledge. Hydrophobic liquids with high O_2_ solubility were also immobilized in porous membranes to fabricate OSMs [35]. Silicone oils were used as the hydrophobic liquid and several substrates were studied. Silicone oils with higher viscosities performed better, which was attributed to lower water vapor permeation rates. Another possibility is the higher viscosity liquids remained immobilized in the substrate pore better. Cells discharged in 20% RH at 0.05 mA cm^−2^ demonstrated capacities between 600–800 mAh g^−1^ C using the OSMs and only 250 mAh g^−1^ with no protection.

MaxPower, Inc. has been developing higher rate OSMs for Li-air batteries with increased power densities. Early work focused on immobilizing hydrophobic liquids with high O_2_ solubilities like perfluorotributylamine, perfluorodecalin, and Krytox 1506 into microporous membranes [26]. Other OSMs were fabricated from gelled FMS123 liquid and silicon rubber [26]. One method to immobilize liquids under development is hardening silicone based liquids via UV curing [27]. For example, a polysiloxane-methacrylate copolymer (Gelest) was prepare by dipping a nonwoven polyphenylene sulfide (PPS) membrane into a mixture of methacryloxypropyle polydimethylsiolxanes and photo initiator and exposing to a UV source with 210–315 nm wavelength with an average power of 1,400 mJ cm^−2^ to crosslink the material. Finished OSMs had thicknesses between 330–1,016 µm. Li-air cell laboratory cells (pouch type, 10 cm^2^) were discharged at a rate of 0.1 mA cm^−2^ at room temperature and 15% RH. The cells with thinner OSMs demonstrated improved discharged capacities of 947 mAh g^−1^ C compared to 786 mAh g^−1^ C for the control cell discharged with no OSM. However, these OSMs were relatively thick limiting O_2_ flux to the air electrode and thus maximum current density. Thinner OSMs (76–89 µm) were fabricated using a RTV-amine (Semicosil 964, Wacker Silicones) coated directly onto an air electrode. The silicone rubber hardened by being moisture cured overnight. Cells discharged with this OSM were able to demonstrate capacities of almost 600 mAh g^−1^ C at a high rate of 0.5 mA cm^−2^. This capacity was three times higher than that demonstrated by the control cell discharged under the same conditions with no OSM.

Difficulties reproducibly fabricating thin, pinhole free OSMs led to the investigation of commercially produced membranes. TCFC, mentioned above, was found to work successfully [18,36]. The membrane is 70 µm thick and has an O_2_ permeability of 240 Barrer. The selectivity at room temperature from air is 2.5–5 mole of O_2_ per mole water vapor depending on the relative humidity. Cells were demonstrated to discharge at rates as high as 0.2 mA cm^−2^, though they failed at rates of 0.5 mA cm^−2^. 100 cm^2^ fixtured cells with dramatically reduced electrolyte quantities discharged for more than 2000 mAh g^−1^ C at a rate of 0.1 mA cm^−2^ in air at 40–50% RH. This was evidence in addition to figure 4 that electrolyte evaporation was slowed to acceptable rates. Pouch type laboratory cells (10 cm^2^) discharged for over 6,000 mAh g^−1^ C in air at 20% RH at a rate of 0.1 mA cm^−2^. 

Sigmund *et al*. [40,41] has reported on superhydrophobic membranes potentially for Li-air cells based on electrospun Teflon AF2400 though it has not been tested in an operating Li-air cell to our knowledge. Teflon AF2400 is an interesting potential OSM material because of its high O_2_ permeability of 990 Barrer and excellent solvent resistance [42]. However, these electrospun membranes may not work well in an actual cell due to the highly porous nature of electrospun mats, leading to problems associated with Knudsen diffusion of water vapor described above.

## 5. Conclusions

OSMs have been demonstrated to successfully allow for ambient operation of Li-air batteries in literature. The desired properties of an OSM are high oxygen permeability, no water vapor permeability, no electrolyte solvent permeability, and no carbon dioxide permeability for secondary applications. In practice, no materials exist with no water vapor permeability, however, materials with higher O_2_ permeation rates (compared to water vapor) can successfully protect the lithium metal anode in a primary Li-air battery. All materials demonstrated as OSMs in the available literature suffer from relatively low O_2_ permeabilities, thus limiting the maximum discharge current density. Therefore novel membranes based on materials with higher O_2_ permeabilities (> 100 Barrer at least) must be developed to increase the power density of Li-air batteries.
